# Investigating the process of evidence-informed health policymaking in Bangladesh: a systematic review

**DOI:** 10.1093/heapol/czz044

**Published:** 2019-06-25

**Authors:** Madeleine Dodd, Rebecca Ivers, Anthony B Zwi, Aminur Rahman, Jagnoor Jagnoor

**Affiliations:** 1 Injury Division, The George Institute for Global Health, UNSW Sydney, 1 King Street, Newtown, Sydney, New South Wales, Australia; 2 School of Public Health & Community Medicine, UNSW; The George Institute for Global Health Australia, UNSW, Australia; 3 Health, Rights and Development (HEARD@UNSW), Faculty of Arts and Social Sciences, School of Social Sciences, University of New South Wales, Sydney, New South Wales, Australia; 4 Centre for Injury Prevention and Research (CIPRB), Dhaka, Bangladesh

**Keywords:** Policy analysis, health policy, evidence-based policy, policy process

## Abstract

Over the last four decades, Bangladesh has made considerable improvements in population health, this is in part due to the use of evidence to inform policymaking. This systematic review aims to better understand critical factors that have facilitated the diffusion of scientific evidence into multiple phases of health policymaking in Bangladesh. To do this an existing policy framework designed by Shiffman and Smith in 2007, was used to extract and synthesize data from selected policy analyses. This framework was used to ensure the content, context and actors involved with evidence-informed policymaking were considered in each case where research had helped shape a health policy. The ‘PRISMA Checklist’ was employed to design pre-specified eligibility criteria for the selection of information sources, search strategy, inclusion and exclusion criteria, and process of data extraction and synthesis. Through our systematic search conducted from February to May 2017, we initially identified 1859 articles; after removal of duplicates, followed by the screening of titles, abstracts and full-texts, 24 articles were included in the analysis. Health policy issues included the following topics: maternal and child health, tobacco control, reproductive health, infectious disease control and the impact and sustainability of knowledge translation platforms. Findings suggested that research evidence that could be used to meet key targets associated with the Millennium Development Goals (MDGs) were more likely to be considered as a political (and therefore policy) priority. Furthermore, avenues of engagement between research organizations and the government as well as collective action from civil-society organizations were important for the diffusion of evidence into policies. Through this article, it is apparent that the interface between evidence and policy formulation occurs when evidence is, disseminated by a cohesive policy-network with strong leadership and framed to deliver solutions for problems on both the domestic and global development agenda.

**Key Messages**

- Framing public health research to align with national and global development priorities can heighten the influence of research on health policy formulation.
- Involving policymakers in the early phase of research production enhances the utilization of research findings in the design and delivery of national programmes.
- Shiffman and Smith’s Policy Prioritization framework is a useful tool to identify why certain health issues attract greater policy prioritization.

## Introduction

Evidence-informed policymaking (EIPM) is the process of using research evidence in health policy to strengthen health systems to benefit the health of the wider population (World Health Organization, 2017). Over the last two decades, EIPM has been emphasized as an essential component for improving population health in high-, middle- and low-income countries (World Health Organization, 2005). Debate around the issue of evidence and policy is widespread and a range of models proposed to understand the interface of evidence and policy have been designed to highlight the differing social, economic and political processes involved with the diffusion of evidence into policy (Bowen and Zwi, 2005). Heightened attention to the issue occurred with the formation of the Alliance for Health Policy and Systems Research in 1999 and later in 2003 the World Health Organization released a report called ‘Knowledge for better health—a conceptual framework and foundation for health research systems’ (Pang *et al.*, 2003; Hanney and González-Block, 2017). Following this the World Health Assembly passed a resolution based on the ‘Mexico Statement on Health Research’. This resolution is centred on how low and middle income country (LMICs) can bridge the ‘know-do gap’ to create sustainable improvements for evidence-informed health systems (World Health Organization, 2005, 2017). Each of these movements are centred on the ‘facilitational model’, whereby policymakers are engaged with research at an early stage so that it is co-produced and more likely to be translated into policy (Cairney and Oliver, 2017)*.* Other models such as the ‘advocacy model’, where evidence is framed in an emotive way to appeal to the ‘irrational’ or ‘emotional’ aspect of policymaking, is given considerably less attention by researchers because of its ethical implications (Cairney and Oliver, 2017). Over the past two decades, greater recognition has been given to the important role qualitative research has in understanding how social, political and economic networks interact to determine how scientific evidence is prioritized and translated into health policies (Saini and Shlonsky, 2012; Redman *et al.*, 2015).

This article has used Buse and colleagues (2012) definition of health policy which is defined as ‘courses of action (and inaction) that affect the set of institutions, organizations, services and funding arrangements of the health system’. Further to this definition the following review has used the ‘heuristic stages model’ to define the different phases involved with health policymaking*.* The ‘heuristic stages model’, clearly demarcates policymaking into the following five stages: ‘agenda setting’, ‘formulation’, ‘adoption’, ‘implementation’ and ‘evaluation’ (Eisenhardt, 1989). Although this theory has been criticized for its linear flow, it does clearly convey the different phases, albeit somewhat over-simplistically, of the policy cycle (Walt *et al.*, 2008). This review aims to identify critical factors involved with the diffusion of evidence during the ‘agenda setting’, ‘formulation’ and ‘implementation’ phase of the policy process, across multiple health issues.

Evidence has been identified as playing a pivotal role in health improvements in Bangladesh since the 1970s (Chowdhury *et al.*, 2013). Despite the extent of poverty, high population density, natural disasters and political instability that Bangladesh endured since independence in 1971, the state has been hailed for its ability to achieve ‘good health at low cost’(Chowdhury *et al.*, 2013). For example, between 2000 and 2010 Bangladesh experienced a 4% per year decline in neonatal mortality, which was nearly double the regional average of 2–2.1% per year (Rubayet *et al.*, 2012). Furthermore in 2010 the United Nations applauded Bangladesh for progress towards achieving Millennium Development Goals (MDGs) 4a and 5a through increased immunization coverage, improved treatment of diarrhoea and decreased fertility rates (Burchett *et al.*, 2012; Larson *et al.*, 2012; Ahmed *et al.*, 2013; Chowdhury *et al.*, 2013). The health improvements listed above have been achieved while per capita spending has remained low in relation to neighbouring countries (Chowdhury *et al.*, 2013).

To highlight the progress in Bangladesh in 2013 ‘The Lancet’ published a special six-part series investigating past achievements and future challenges for the country. This series emphasized communicable disease control and family planning as two areas in which Bangladesh has experienced much of its success (Chowdhury *et al.*, 2013). This is evident through the significant decline in infectious diseases; in 1986 communicable diseases accounted for 52% of all deaths in Bangladesh—whereas in 2006 this proportion had declined to 11% (Ahsan Karar *et al.*, 2009). This decline in communicable diseases has caused an epidemiological shift where non-communicable diseases now account for the highest burden of disease (68%) (Ahsan Karar *et al.*, 2009). Close investigation about what has led to scientific evidence being used to inform policy formulation and implementation, could assist researchers in identifying strategies to prioritize issues that pose a major public health threat. Literature surrounding the diffusion of evidence into health policy, highlights the important role individuals, interests and ideas have in determining how evidence is used in health policy (Walt *et al.*, 2008). To explore factors that enhance the diffusion of evidence into policy, Shiffman and Smith’s (2007) policy prioritization framework has been used to underpin the data synthesis in this review. Although this framework was not designed explicitly for analysing the translation of research evidence into policy, it incorporates four components to understand how evidence can be produced and framed to facilitate its diffusion into health policies. These core elements are centred around the power of the people advocating for the health issue, the power of framing evidence to resonate with national and global priorities, the impact political and societal values have on how evidence is or is not used, and how the severity of an issue can determine political prioritization and action (Shiffman and Smith, 2007). Variation in these four domains can influence why certain issues that have been underpinned by evidence can lose political support, as well as identifying key factors that facilitate sustained interest in a health issue (Shiffman and Smith, 2007).

## Methods

The following methods were structured in accordance to the ‘Preferred Reporting Items for Systematic review and Meta-analysis’ (PRISMA) (Moher *et al.*, 2009). The PRISMA checklist is an appropriate tool for systematic reviews designed to appraise and synthesize qualitative data related to health policy. This is evidenced through its past application to a systematic review investigating the barriers and facilitators surrounding the use of evidence by policymakers published by Oliver and colleagues in 2014 (Oliver *et al.*, 2014). The PRISMA checklist ensures that systematic reviews are designed and reported on in a comprehensive way. A protocol for this review was not published, but the review had a pre-defined search strategy with clearly defined inclusion criteria. In addition to PRISMA guidelines this review is also in accordance with the ‘Enhancing transparency in reporting the synthesis of qualitative research’ statement (ENTREQ) designed by Tong and colleagues (2012).

Traditionally, systematic reviews have been designed to report quantitative results by critically appraising and reporting the rigour of statistical evidence and transparently reporting the findings to help inform policymakers and clinicians about the evidence base surrounding interventions (Lavis *et al.*, 2005; O’brien *et al.*, 2014). Greater attention to the systematic synthesis of qualitative data has emerged over the years to better understand how contextual influences and social and political interactions affect different elements of policymaking and policy formulation in real-world situations, rather than controlled experiments (O’brien *et al.*, 2014). Qualitative synthesis is particularly important in the field of policy where individual, political and economic factors play a pivotal role in how evidence is used in the health policy process (Lavis *et al.*, 2005). This review uses qualitative data to identify critical factors that have facilitated the diffusion of evidence into policy formulation in Bangladesh.

This study consisted of three main steps: (1) Systematic searching for relevant studies, using predetermined search terms and information sources; (2) Study appraisal and data extraction; and (3) Deductive synthesis of extracted data using themes derived from Shiffman and Smith’s Policy Prioritization framework.

### Eligibility criteria

#### Inclusion and exclusion criteria

This review selected literature based on the following criteria:

- Peer-reviewed primary studies (no restrictions on study design);
- Published after the year 2000;
- Explored the use of evidence in policymaking as well as the implementation and scale-up of health policies;
- Multi-country studies were selected if they included Bangladesh; and
- Mixed-method studies, only the qualitative aspect of the study was synthesized.

The decision to include studies published after the year 2000 was made after an initial search using the Medline database identified no literature surrounding EIPM in Bangladesh, with the exception of an opinion piece discussing the reduction of cholera in Bangladesh in the 1980s. Furthermore, heightened attention surrounding the importance of health policy research for LMICs occurred in 2003 with the publication of ‘Knowledge for a Better World’ (Pang *et al.*, 2003; Hanney and González-Block, 2017). Leading to a rise in scholarly interest in the topic.

#### Information sources

The following databases were searched for eligible primary studies between February and May 2017: PubMed; Medline; Scopus; Cochrane Library; International Initiative for Impact Evaluation; and Campbell International Development Collaboration.

Prior to the formal search, a broad review of literature surrounding the diffusion of research evidence into health policy from both high- and low-income countries was synthesized to identify key terms. Once a list of key terms was constructed, a consultation between the first author and a public health librarian tested the terms to evaluate the relevance of the search results. This was done by screening the titles of the first 20 papers returned through each search. After this process, a combination of the following search terms that included programmes within Bangladesh where evidence has influenced health policy formulation were used to search the above databases: “policy” [MeSH Terms] OR “policy” [All Fields] AND Health Promotion/AND Policy Making/AND health policy reform AND ‘knowledge translation’ AND Change Theory AND “communication” [MeSH Terms] “communication” [All Fields] AND Research/AND Bangladesh.mp (short for multi-purpose). or Bangladesh/. After the first search, results were shared with the fourth author (AR) who suggested including the following diseases that have been reduced through the application of evidence-informed policymaking: “Expanded Programme on Immunisation” OR “Diarrhoeal disease control” OR “Lymphatic filariasis” OR “Malaria” OR “Tuberculosis”.

#### Data synthesis

All search results were stored in EndNote 7. This review used a form of deductive analysis referred to as ‘framework analysis’, where themes are derived from existing literature (Barnett-Page and Thomas, 2009). Framework analysis is recommended for the field of health policy to firstly test and strengthen existing policy frameworks and secondly to ensure the analysis is done in a structured way that considers the role of actors, policy content, the political and economic context and the policy process (Walt *et al.*, 2008; Dixon-Woods, 2011). The framework employed for this analysis was Shiffman and Smith’s (2007), policy prioritization framework. The benefit of Shiffman and Smith’s framework is that it has been designed specifically for identifying how health issues are prioritized in LMICs and therefore considers the influence that donor organizations and external funding have on policy decisions (Shiffman and Smith, 2007). Walt and Gilson (2014) identified this framework as being one of the most well-developed policy prioritization frameworks designed for health issues.

The framework consists of four broad categories which include: ‘Actor Power’, ‘Ideas’, ‘Political Contexts’ and ‘Issue Characteristics’. These categories are broken down further into 11 factors that provide more specific sub-categories that are required to shape political prioritization. These factors include: (1) Policy Community Cohesion, (2) Leadership, (3) Guiding institutions, (4) Civil society mobilization, (5) Internal frame, (6) External frame, (7) Policy windows, (8) Global governance structure, (9) Credible indicators, (10) Severity and (11) Effective interventions (Shiffman and Smith, 2007). See Table 1 for further details. Although this framework is designed to assess the ‘political prioritisation’ or ‘agenda-setting phase’ of a health issue, the various categories made it possible to apply the framework to articles that were focused on areas of policymaking outside the agenda setting phase. Prior to the synthesis of each article a template was created in Excel, consisting of the 4 categories and 11 factors that make up Shiffman and Smith’s Framework.

**Table 1:** Shiffman and Smith’s (2007, p. 1371) Policy Prioritization Framework

Category	Description	Factors shaping political priority
Actor power	The strength of the individuals and organizations concerned with the issue	1. Policy community cohesion: the degree of coalescence among the network of individuals and organizations that are centrally involved with the issue at the global level
		2. Leadership: the presence of individuals capable of uniting the policy community and acknowledged as particularly strong champions for the cause
		3. Guiding institutions: the effectiveness of organizations or co-ordinating mechanisms with a mandate to lead the initiative
		4. Civil society mobilization: the extent to which grassroots organizations have mobilized to press international and national political authorities to address the issue at the global level
Ideas	The ways in which those involved with the issue understand and portray it	5. Internal frame: the degree to which the policy community agrees on the definition of, causes of, and solutions to the problem
		6. External frame: public portrayals of the issue in ways that resonate with external audiences, especially the political leaders who control resources
Political contexts	The environments in which actors operate	7. Policy windows: political moments when global conditions align favourably for an issue, presenting opportunities for advocates to influence decision-makers
		8. Global governance structure: the degree to which norms and institutions operating in a sector provide a platform for effective collective action
Issue characteristics	Features of the problem	9. Credible indications: clear measures that show the severity of the problems and that can be used to monitor progress
		10. Severity: the size of the burden relative to other problems, as indicated objective measures such as mortality levels
		11. Effective interventions: the extent to which proposed means of addressing the problems are clearly explained, cost effective, backed by scientific evidence, simple to implement and inexpensive

Each article was assessed for quality using the ‘Mixed Methods Appraisal Tool’, this tool has a component which is specific for assessing the quality for qualitative studies (McGill University, 2011). In the review, quality appraisal was used to facilitate a deeper understanding of the included studies and was not used to exclude studies. Therefore, the MMAT was a useful tool to identify the study design and to critically appraise the coherence between data sources, collection, synthesis and interpretation (Hong *et al.*, 2018).

Prior to the data extraction process, codes were derived from Shiffman and Smith’s Policy Prioritization 2007 framework. The following data were extracted by the first author (MD) from the 24 eligible articles:

- Country/countries in which the study was undertaken (particularly important for multi-country studies);
- Study design;
- Number of participants included in the study and the type of data collection tools (e.g. in-depth interviews and/or focus group discussions);
- Theoretical basis of the study; and
- Any data relating to the 11 factors outlined in Shiffman and Smith’s framework. If data related to the use of evidence in policymaking, but was not captured in Shiffman and Smith’s framework, it was also extracted.

After the initial synthesis definitions and coding were reviewed by the last author (JJ) and any disagreements were discussed and alterations to synthesis were made accordingly.

On completion of the initial synthesis the studies were coded a second time by the first author to ensure factors relevant to the diffusion of evidence into policymaking were captured.

## Results

**Figure 1.**
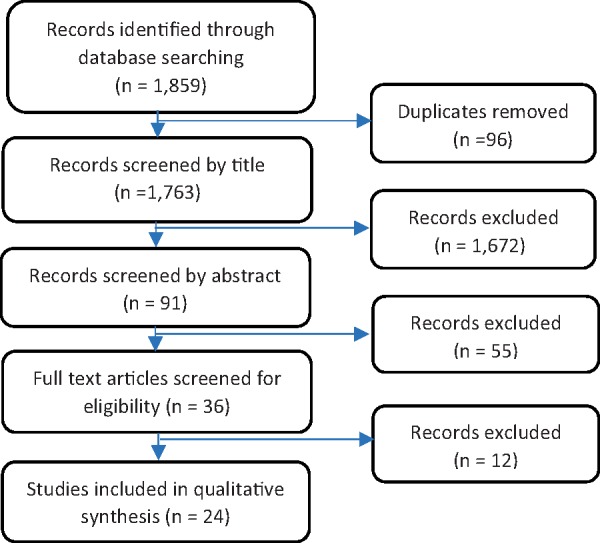
PRISMA flow diagram illustrating the systematic search of literature for this review.

After the returned articles were screened for duplicates and then by title, abstract and inclusion criteria, 24 articles were selected. This process is illustrated in Figure 1, PRISMA Flow Diagram. Out of the 24 papers identified 16 were qualitative and 8 used mixed methods. The large majority were case studies focusing either prospectively on the process of policy formulation or retrospectively analysing factors that contributed to the application of research into policy. Of the selected studies 10 were based in Bangladesh, while the other 14 were multi-country studies that included Bangladesh alongside other countries of Asia, Africa and South America. A myriad of health and knowledge translation topics were addressed with studies in Bangladesh exploring the scaling up of electronic health records; neonatal and maternal health; health research utilization by the Government of Bangladesh; tuberculosis control programmes; the large-scale implementation of nutrition programmes; the scaling up of zinc for young children; The Saving of Newborn Lives Program; and immunization coverage (Shiffman and Smith, 2007; Larson *et al.*, 2012; Pelletier *et al.*, 2012; Rubayet *et al.*, 2012; Ashraf *et al.*, 2015; Walugembe *et al.*, 2015). Multi-country studies centred on the evaluation of stakeholder dialogues; the impact of Knowledge Translation Platforms based in LMICs; outcomes of donor initiated programmes; and smokeless tobacco control programmes (Behague *et al.*, 2009; Bennett *et al.*, 2012; El-Jardali *et al.*, 2014; Jackson-Morris *et al.*, 2015; Shroff *et al.*, 2015; Norton *et al.*, 2016; Teerawattananon *et al.*, 2016). Although the latter topics do not focus exclusively on a specific policy issue, they are useful at providing insights into the reality of the process involved with EIPM and the availability and use of evidence by both researchers and policymakers. The search results are available as a Supplementary File and present: the title, study-type, theory and/or framework used, health issue and main outcomes for each study.

The following results have been structured under each of the four categories identified by Shiffman and Smith and their 11 factors. Although not every paper was about ‘policy prioritisation’ or ‘agenda setting’, the framework facilitated comparison of characteristics between the health issues presented in the literature.

### Category 1: Actor power

#### Policy community cohesion

Cohesion between key stakeholders involved with an issue was highlighted as being instrumental for the political prioritization, implementation and scale-up of policies (Mannan, 2003; Jahan, 2007; Behague *et al.*, 2009; Balabanova *et al.*, 2013; Bowser *et al.*, 2014; Jackson-Morris *et al.*, 2015). A small group of actors concerned with newborn health called the ‘Newborn Working Group’ played an important role in facilitating the formulation of evidence-informed policies to improve newborn health (Shiffman and Sultana, 2013). This group designed training modules that were later taken up by the government and influenced the Ministry of Health and Family Welfare to include newborn health in one of its five priorities in the 2002 ‘Management for Childhood Illness Strategy’ (Shiffman and Sultana, 2013). In addition to the political prioritization of issues, Jackson-Morris emphasize the important role local level taskforces had in implementing policies designed to decrease the use of tobacco(Jackson-Morris *et al.*, 2015). Although these taskforces were district level actors, they were encouraged to attend national meetings which enhanced their capacity to implement specific regulations while strengthening tobacco control policies by sharing information (Jackson-Morris *et al.*, 2015). District and sub-district policy actors play an important role in the scale-up of health policies within Bangladesh and therefore their inclusion within these policy networks is important (Balabanova *et al.*, 2013).

#### Leadership

The importance of an individual leader to promote a health issue was cited in the literature that analysed the prioritization of newborn health (Rubayet *et al.*, 2012). In this case, prominent paediatricians were identified as the national champions who increased awareness and advocated for evidence-based solutions to be integrated into national policy (Rubayet *et al.*, 2012; Shiffman and Sultana, 2013).

#### Guiding institutions

An institution or committee within a larger organization was often a key factor for the prioritization and implementation of multiple health policies (Jahan, 2007; Burchett *et al.*, 2012; Rubayet *et al.*, 2012; Khan *et al.*, 2014; Shroff *et al.*, 2015). An analysis of policies that led to improved population health and health systems, identified the establishment of institutions in local, regional and national levels of government as an important contributor to the large-scale implementation of policies (Balabanova *et al.*, 2013). For example, the Directorate General of Health Services and the Ministry of Health and Family Welfare were identified as being essential for the initiation and process of policy formulation (Rubayet *et al.*, 2012; Shiffman and Sultana, 2013; Uddin *et al.*, 2013; Ashraf *et al.*, 2015; Teerawattananon *et al.*, 2016). Electing a programme manager for the newborn health programme within the government, coupled with the Ministry of Health and Family Welfare’s release of the ‘National Neonatal Health Strategy’ were considered as vital components for the nationwide scale-up of newborn health services (Rubayet *et al.*, 2012; Shiffman and Sultana, 2013). Likewise, the translation of evidence to policy surrounding the use of zinc as a treatment for childhood diarrhoea was facilitated by the establishment of a ‘National Advisory Committee’. This Committee acted as the linkage between policymakers, researchers and clinicians (Larson *et al.*, 2012; Panisset *et al.*, 2012).

In addition to national institutions, guidelines from the World Health Organization were important for guiding the development of specific policies. For example, the use of zinc for the treatment of diarrhoea was aided by a half day course led by the World Health Organization that reviewed the current zinc guidelines (Larson *et al.*, 2012). Furthermore, reproductive health interventions outlined in the ‘Health and Population Sector Plan’ aligned with those from the World Health Organization. This alignment helped with the longer-term sustainability of the interventions included within the strategy (Jahan, 2007). Other important organizations that influenced what was considered a priority in Bangladesh included ‘The United Nations Children’s Fund (UNICEF), Global Vaccine Alliance (GAVI), The World Bank, The Asian Development Bank, USAID and The Gates Foundation’ (Shiffman and Sultana, 2013; Uddin *et al.*, 2013; Teerawattananon *et al.*, 2016).

#### Civil society mobilization

Multiple studies discussed the impact local non-governmental organizations and advocacy groups can have on the diffusion of scientific evidence into government policy (Mannan, 2003; Rubayet *et al.*, 2012; Shiffman and Sultana, 2013). The increased priority of newborn health in Bangladesh was attributed to a local paediatrician who successfully mobilized physicians across Bangladesh to lobby the government. In addition, the presentation of national data by local advocates to policymakers was considered effective in generating policy action (Rubayet *et al.*, 2012; Shiffman and Sultana, 2013). Civil society groups were considered important during the implementation phase of a new family planning, maternal and child health intervention to enhance the programmes sustainability and safeguard the intervention from collapsing as a result of political changes (Jahan, 2007; Balabanova *et al.*, 2013). Local researchers were identified as playing a vital role in advocacy efforts and the research organization, the International Centre for Diarrhoeal Disease, Bangladesh (ICDDR,B) were mentioned in multiple publications (Pelletier *et al.*, 2012; Rubayet *et al.*, 2012). The Bangladesh Rural Advancement Committee (BRAC) was also noted as being essential in collaborating with the government to provide health services that the government could not (Balabanova *et al.*, 2013). Furthermore, civil-society organizations were often referred to as an ‘extension of government services’. A major contribution BRAC has made to the countries health system is through the provision of village health workers who have reached up to 110 million people (Balabanova *et al.*, 2013). To enhance the collaboration between government and civil-society organizations in 1998 the Bangladesh ‘Sector Wide Approach program’ (SWAp) was established (Balabanova *et al.*, 2013). The SWAp collated 120 health sector programmes with the aim of reducing programme and financial duplication (Balabanova *et al.*, 2013).

### Category 2: Ideas

#### Internal frame

A recurring topic from the literature was the importance of policymaker engagement with the process of research (Burchett *et al.*, 2012; Larson *et al.*, 2012; Pelletier *et al.*, 2012; Rubayet *et al.*, 2012; Shiffman and Sultana, 2013; Uddin *et al.*, 2013; El-Jardali *et al.*, 2014; Ashraf *et al.*, 2015; Shroff *et al.*, 2015; Walugembe *et al.*, 2015; Norton *et al.*, 2016). Details of methods of engagement varied between studies. Multi-stakeholder dialogues were the most commonly referenced strategy that helped construct definitions and possible strategies for the health problem. A variety of terms were used to describe multi-stakeholder dialogues, these included ‘Deliberative Dialogues’(El-Jardali *et al.*, 2014) ‘Focusing Events’(Shiffman and Sultana, 2013), and ‘Information Sharing Sessions’(Larson *et al.*, 2012). Despite the interchangeable titles the overarching purpose of multi-stakeholder dialogues was to encourage policymakers, researchers and important stakeholders such as donors, national professional bodies and representatives from different ministries to share and negotiate on knowledge and ideas surrounding certain evidence and formulate plans of action (Mannan, 2003; Larson *et al.*, 2012; Ahmed *et al.*, 2013; Ashraf *et al.*, 2015). Stakeholder collaboration was identified as being an integral strategy to utilize, before, during and on completion of the study. Having constant engagement with policymakers was reported to strengthen the relationships between the researchers and relevant ministries (Walugembe *et al.*, 2015). Two studies identified how consistent stakeholder engagement activities facilitated the uptake of Misoprostol into recommended interventions for government health workers during the management of postpartum haemorrhages (Walugembe *et al.*, 2015; Norton *et al.*, 2016).

Multi-stakeholder dialogues were identified as being important for individuals with differing perspectives to come to a common agreement and take collaborative action to solve a problem (Pelletier *et al.*, 2012; Hawkes *et al.*, 2016). In order to achieve the objectives of the multi-stakeholder dialogues intricate planning was recommended to firstly ensure that stakeholder views are captured and that each participant has an equal opportunity to express their opinion. It was recommended that each participant be interviewed early on with insights from these interviews to inform the topics and activities of the dialogue. This technique is called the ‘Mutual Gains’ approach and aims to provide all participants with an equal voice in order to have tangible actions after the meeting (Ashraf *et al.*, 2015). Furthermore, stakeholder mapping was suggested as a planning technique that could be used to ensure energy is channelled to the relevant stakeholders (El-Jardali *et al.*, 2014).

#### External frame

Having a strong external frame surrounding a health issue was integral for mobilizing support from policymakers and international organizations who were not already involved with the issue (Walugembe *et al.*, 2015). Academic publications, conferences and seminars were considered important in raising the profile of an issue and areas for action (Jahan, 2007; Shiffman and Sultana, 2013). In addition to these traditional forms of dissemination, the SUZY programme (scaling up of Zinc for the treatment of diarrhoea) disseminated twice yearly project newsletters to 20 000 people and together with the Ministry of Health and Family Welfare constructed a website with evidence-based information about the use of zinc for diarrhoea (Larson *et al.*, 2012). The papers focusing on nutrition identified mass media in the form of a front-page newspaper article, as the catalyst that increased the political prioritization of childhood nutrition (Pelletier *et al.*, 2012; Sanghvi *et al.*, 2016). Furthermore, mass media in Bangladesh was identified as being a method that could be used to hold policymakers to account for deficits in political action to improve health outcomes (Balabanova *et al.*, 2013). Three authors noted the importance of generating strong internal collaboration to influence external audiences (Jahan, 2007; Bowser *et al.*, 2014; Walugembe *et al.*, 2015).

### Category 3: Political contexts

#### Policy windows

Evidence surrounding newborn mortality in Bangladesh, political leadership, an evidence-based strategy and increased funding, were essential factors that opened a policy window for the Saving of Newborn Lives programme (Shiffman and Sultana, 2013). In 2000, The Gates Foundation provided Save the Children USA with $50 million to fund improvements in newborn health, Bangladesh was one of the six countries selected by Save the Children for this programme (Shiffman and Sultana, 2013). These resources enabled local newborn researchers to disseminate evidence about the high burden of newborn mortality and highlight the issue as a barrier to achieving MDG 4 (Shiffman and Sultana, 2013). The availability of funding from GAVI was considered a major driver for the introduction of new vaccines (Burchett *et al.*, 2012). The influence of GAVI resources also had a distorting impact and occasionally had precedence over local priorities as highlighted by Burchett (Burchett *et al.*, 2012). Furthermore, diminished funding was linked to the demise of the Health Economic Institute which played a role in health policy analyses (Bennett *et al.*, 2012).

Disease outbreaks, such as H1N1 was an opportune time to introduce a new vaccine. Uddin *et al.* (2013) mentioned the pressure policymakers were under due to mass media coverage of the H1N1 outbreak as an important reason for why the Ministry of Health and Family Welfare acted so quickly to introduce a new vaccine. This highlights the role mass media has in creating policy windows, while also illustrating the strong influence public opinion has on decisions that are made at the ministerial level.

### Global governance structure

Global frameworks such as the MDGs that hold signatory states accountable for their success were frequently cited as providing windows for evidence to be translated into policy (Behague *et al.*, 2009; Shiffman & Sultana, 2013; Ashraf *et al.*, 2015). Not only did the MDGs have the ability to influence what polices were prioritized, but they also provided researchers with the ability to justify how the translation of their evidence into policies could achieve or maintain country progress for meeting the MDGs (Burchett *et al.*, 2012; Ashraf *et al.*, 2015). After a body of evidence released by The Lancet called ‘The Neonatal Series’ indicated that the failure to improve newborn health would impede government efforts to achieve MDG 4, many local and government organizations from within Bangladesh began to mobilize to improve newborn (infants <28 days) mortality rates (Rubayet *et al.*, 2012; Shiffman and Sultana, 2013)*.* Furthermore, global statements such as ‘stalled progress’ to achieving the MDGs were identified as influences that led to increased attention for the ‘Mainstreaming of Nutrition Initiative’(Pelletier *et al.*, 2012).

Although the MDGs and SDGs were noted to have contributed to increased government uptake of EIPM, one publication suggested that the global pressures to achieve the MDGs often meant that health problems that were a local but not global priority were neglected (Behague *et al.*, 2009).

Although a lot of emphasis was given to the positive impact that can occur through the work of the Ministry of Health and Family Welfare and Directorate General Health Services, political instability and the re-election of a new government was identified as having a detrimental effect to the policy formulation process (Bennett *et al.*, 2012; Larson *et al.*, 2012; Rubayet *et al.*, 2012; Shiffman and Sultana, 2013; El-Jardali *et al.*, 2014). Government re-election was stated to lead to decreased funding provided to knowledge translation platforms such as the Health Economic Institute which reduced their capacity to produce systematic reviews and policy briefs (Bennett *et al.*, 2012; El-Jardali *et al.*, 2014). Furthermore, government changeover was considered as a significant challenge for the Saving of Newborns Lives programme, because there was no guarantee that the new government would continue the work of the former ministry (Shiffman and Sultana, 2013).

## Category 4: Issue characteristics

### Credible indications

Credible data and evidence surrounding interventions were considered as a driving factor for EIPM in three of the papers (Rubayet *et al.*, 2012; Shiffman & Sultana, 2013; Hawkes *et al.*, 2016; Teerawattananon *et al.*, 2016). Reluctance to act on international evidence was demonstrated during the establishment of the Saving of Newborn Lives programme (Rubayet *et al.*, 2012; Shiffman and Sultana, 2013). Evidence generated through the efforts of local advocacy groups to show the lack of knowledge about newborn health coupled with statistics from the 1999–2000 Demographic Health Survey were represented as being crucial for gaining support from policymakers (Shiffman and Sultana, 2013). Access to credible local data was identified as a barrier for Knowledge Translation Platforms when they attempted to influence policy (El-Jardali *et al.*, 2014). This was also the case for the Mainstreaming Nutrition Initiative, the release of local data about the burden of disease of both anaemia and stunting in children was considered a crucial element for increasing efforts to improve childhood nutrition (Pelletier *et al.*, 2012). In addition to burden of disease data policymakers also wanted simple evidence-based interventions that could be applied (Pelletier *et al.*, 2012).

### Severity

Burden of disease data was the most commonly cited type of data that generated political interest in a health issue (Burchett *et al.*, 2012; Pelletier *et al.*, 2012; El-Jardali *et al.*, 2014; Khan *et al.*, 2014; Sanghvi *et al.*, 2016). Statistics revealing that 92% of infants and 68% of school-aged children in Bangladesh had anaemia was crucial in gaining political attention for the prevention of anaemia (Pelletier *et al.*, 2012). Likewise, data surrounding neonatal mortality and the prevalence of certain infections such as rubella and pneumococcal played a significant role in the formulation of the newborn national strategy and the expanded immunization programme (Burchett *et al.*, 2012). Health conditions that did not demonstrate the severity of the health issue were often not provided with the same amount of support than those that did. Difficulty collecting prevalence data about the use of Smokeless Tobacco meant that there was a major knowledge gap in their campaign to capture political attention (Khan *et al.*, 2014).

### Effective interventions

Articulating possible solutions to address a health issue was an essential factor contributing to the formulation and implementation of many health policies (Jahan, 2007). Policy briefs were cited frequently as a method to succinctly describe the situation, solution and cost-effectiveness of a policy (Uddin *et al.*, 2013; El-Jardali *et al.*, 2014; Shroff *et al.*, 2015). Emphasis was given to the positive outcome a two-page policy brief about a H1N1 vaccine, that outlined why the intervention was necessary (Shroff *et al.*, 2015). Furthermore, the article argued that the effectiveness of this brief was strengthened by the pre-existing relationship between researchers and policymakers (Shroff *et al.*, 2015). Systematic reviews were not explored in detail in any of the articles, but were mentioned as being tools that can aid policymakers in having a summary of the latest evidence surrounding specific topics. It was however noted that KTPs and researchers often released systematic reviews and policy briefs with a large amount of variation that included limited local evidence (El-Jardali *et al.*, 2014).

Field visits to sites where the research programme of interest is being implemented was an effective strategy of sharing evidence-based programme activities with stakeholders (Rubayet *et al.*, 2012; Shiffman and Sultana, 2013; Norton *et al.*, 2016). During these visits, key stakeholders from the ministry would be present. Shiffman and Rubayet both linked the development of the 2009 ‘National Neonatal Health Strategy’ with a field visit to Nepal which demonstrated what was involved with a comprehensive neonatal health government strategy and how this can be applied (Rubayet *et al.*, 2012; Shiffman and Sultana, 2013). Interestingly cost-effectiveness was only cited in two publications two publications listed programme feasibility as an important component for EIPM. Uddin *et al.* (2013) mentioned the importance of cost-effectiveness information in the initial policy dialogues surrounding vaccines. Both multi-country publications exploring the impact of Knowledge Translation Platforms indicated that the ongoing investment required for these institutes meant that Knowledge Translation Platforms were often underfunded and therefore could not achieve their main function which was to provide the government with evidence-based policy recommendations (Bennett *et al.*, 2012; El-Jardali *et al.*, 2014). Interestingly, in the case of the introduction of new vaccines and the launch of the Mainstreaming of Nutrition Initiative, cost-effectiveness studies were not considered as a crucial element for their introduction and data about disease severity was considered to be more significant (Burchett *et al.*, 2012; Pelletier *et al.*, 2012). In the previous section Teerawattananon *et al.*, (2016) mentioned that economic evaluations were often not used in Bangladesh because the data were not sourced locally, this point that was previously made could explain why programme feasibility was not identified as a major factor.

## Discussion

Overall, 24 articles were identified and summarized to better understand factors facilitating the diffusion of evidence into policymaking within Bangladesh. The most important findings through the deductive analysis included the importance of early multi-sectoral dialogue between research producers and necessary stakeholders, the coherent and consistent framing of an issue and its solutions and the high level of influence global governance frameworks, such as the MDGs have on national policy agendas. Furthermore, the role of development assistance targeting specific goals outlined in these frameworks was identified as a major facilitator for the diffusion of evidence in government policies.

Multiple theoretical frameworks were used to guide the policy analysis that occurred in the selected articles. These frameworks consisted of both knowledge translation and policy priority setting models (Behague *et al.*, 2009; Pelletier *et al.*, 2012; Rubayet *et al.*, 2012; Shiffman and Sultana, 2013; El-Jardali *et al.*, 2014; Shroff *et al.*, 2015; Norton *et al.*, 2016). Graham’s Knowledge to Action Framework was used to analyse the exchange of evidence during multi-stakeholder dialogues and is a useful theoretical framework that can be applied to plan and evaluate the outcomes of these types of meetings (Norton *et al.*, 2016). Shiffman and Smith’s (2007) Framework was used and refined by Shiffman and Sultana (2013) during the analysis of the Saving of Newborn Lives programme to be specific for Bangladesh. Other frameworks such as Jacobson’s framework for knowledge translation, which highlights five categories: user groups, the issue, the research, the researcher–user relationship and dissemination strategies (Jacobson *et al.*, 2003) were also referenced. Like Graham’s framework, this theoretical work looks primarily at the exchange of evidence to policymakers. Shiffman and Smith’s model has a greater emphasis on how to get a topic onto the policy agenda and although it mentions the importance of credible indicators, the framework is not entirely focused on evidence translation (Shiffman and Smith, 2007). Despite differences in the purpose of the cited theories, they help provide an understanding of what and how the pathway from evidence to policy can be supported.

A common theme captured through this review is that robust evidence alone in unlikely to influence policy and must be combined with well-orchestrated methods for dissemination (Bowen and Zwi, 2005; Shroff *et al.*, 2015). Multi-stakeholder dialogues and established avenues of engagement between researchers and policymakers was a technique that aided the formulation of cohesive policy networks (Shiffman and Smith, 2007). This cohesion aided the success of evidence-based policy formulation that improved newborn survival rates, childhood nutrition and led to the introduction of new vaccines (Burchett *et al.*, 2012; Pelletier *et al.*, 2012; Rubayet *et al.*, 2012; Uddin *et al.*, 2013; Shroff *et al.*, 2015). ‘Internal Frame’ features as Category 5 of Shiffman and Smith’s (2007) Framework and is defined as ‘the degree to which the policy community agrees on the definition of, causes or and solutions to the problem’. Essential to the formulation of an internal frame is the level of coalescence within the policy networks driving the issue forward. A strategy that can help foster a cohesive community are stakeholder engagement activities, this corresponds with many knowledge translation frameworks. For example, Lavis *et al.* outlines the fostering of a supportive culture between the two communities (policymakers and researchers) in the first category of his knowledge translation framework (Wilson *et al.*, 2013). This literature suggests that the relationship the non-government research organization ICDDR,B has with the Ministry of Health and Family Welfare has played a significant role in transferring evidence into the formulation of policies in Bangladesh (Pelletier *et al.*, 2012; Shiffman and Sultana, 2013). Overall these cases illustrate that a vital element of EIPM is ensuring that policy and research communities are working in collaboration rather than in isolation (Caplan, 1979)*.* Initiatives such as the Bangladesh Sector-Wide Approach programme helped to facilitate the collaboration between private and public institutions and highlights the significant role non-government and civil society organizations play within Bangladesh’s health system (Balabanova *et al.*, 2013).

Having local data was also seen to be important. Both policymakers, non-government organizations and civil society groups were most likely to take action when they were presented with credible data collected from Bangladesh (Burchett *et al.*, 2012; Pelletier *et al.*, 2012; Rubayet *et al.*, 2012; Shiffman and Sultana, 2013). Multiple nation-wide health surveys that revealed the burden of disease for newborn mortality rates, childhood anaemia and the incidence of Haemophilus Influenza Type B (Hib) were used by civil society groups to justify why evidence-based solutions needed to be delivered at scale (Pelletier *et al.*, 2012; Rubayet *et al.*, 2012; Shiffman and Sultana, 2013). In the absence of local evidence surrounding the effectiveness of a policy, field visits were a useful strategy that could be deployed to bridge this knowledge gap and showcase the impact of an evidence-based policy in a real-life scenario (Rubayet *et al.*, 2012; Shiffman and Sultana, 2013). Civil society groups that had the most impact on policymakers were those consisting of paediatric clinicians and well respected research institutes like ICDDR,B (Larson *et al.*, 2012; Rubayet *et al.*, 2012; Shiffman and Sultana, 2013). The importance of both relevant data and who is presenting the data was outlined in Shiffman and Lavis’s policy prioritization and knowledge translation frameworks (Shiffman and Smith, 2007; Wilson *et al.*, 2013). Although neither of these frameworks specify what credible data are, the literature reviewed suggests that national data about disease severity can be a persuasive tool to generate support from advocacy groups to ultimately gain the attention of policymakers (Burchett *et al.*, 2012). Interestingly, cost-effectiveness studies while seen as important in relation to the zinc study were not commonly cited. This was perhaps a result of the gap in the availability of cost-effectiveness information from Bangladesh (Teerawattananon *et al.*, 2016).

An overarching influence that featured in the cases that successfully integrated evidence into policy was the impact of global trends and donor funding (Burchett *et al.*, 2012; Pelletier *et al.*, 2012; Rubayet *et al.*, 2012; Shiffman and Sultana, 2013). MDG 4a (reducing child mortality by two-thirds), and MDG 5a (reducing maternal mortality by two-thirds) were continuously referenced as a motivating factor that increased government commitments to improve newborn health and decrease childhood nutrition, and the MDGs were also classified as ‘internationally-endorsed evidence based policy-making’ (Behague *et al.*, 2009; Pelletier *et al.*, 2012; Rubayet *et al.*, 2012; Shiffman and Sultana, 2013). In the case of the Saving of Newborn Lives, global and national advocates highlighted that without taking action to improve newborn health, Bangladesh would fail to achieve MDG 4 (Rubayet *et al.*, 2012). Many informants mentioned that the need to prove progress towards achieving the MDGs, meant that funding and research activities were directed to programmes that could report this evidence (Behague *et al.*, 2009). Furthermore, the push to create ‘internationally-endorsed evidence-based policies’ was also coupled with significant funding (Shiffman and Sultana, 2013; Uddin *et al.*, 2013), which was considered as being a major reason for why the Government of Bangladesh prioritized certain programmes (Behague *et al.*, 2009; Burchett *et al.*, 2012; Uddin *et al.*, 2013). Although the literature gave reference to the MDGs and the aims of donor organizations overpowering national and local priorities (Behague *et al.*, 2009), the MDGs did successfully persuade governments to embed evidence-based practices into national policies (Burchett *et al.*, 2012; Pelletier *et al.*, 2012; Rubayet *et al.*, 2012; Shiffman and Sultana, 2013).

### Strengths and limitations

This review looks at both activities that have the potential to translate evidence, e.g. multi stakeholder dialogues, as well as overarching themes that lead to the scale-up and formulation of nation-wide policies.

An important limitation of this study is that it drew only on peer-reviewed literature. However, it is unlikely that detailed policy analysis of relevant policy processes were published in grey literature. However, some such studies may have occurred and would have been missed. Furthermore, many of the studies selected for this review are related to programmes that were focused on achieving MDG 4a or MDG 5a. An analysis of a policy formulation process that is not related to an MDG-focus condition would provide valuable insight into how policy is formed without the expectations and influences from global organizations. Furthermore, this review included only studies published in English and therefore may have overlooked policy analyses in Bengali.

## Conclusion

This review highlights that evidence is just one necessary component required to influence policy in Bangladesh. While necessary and vital, credible research is not sufficient to drive EIPM, and many other factors are important. Through this review enhancing the interface of the policy and researcher communities is an essential activity that has the ability to address conflicting values to ultimately facilitate the formulation of EIPM. Furthermore, this review emphasizes that engagement should be undertaken early on, in the initial stages of the research programme. In relation to EIPM in a LMIC setting, the impact of donor funding and linking evidence to global frameworks must be appreciated. Further investigation into the long-term sustainability of the programmes would be helpful to better understand the implications of donor funding and the need for further investigations surrounding cost-effective studies.

## Ethical approval

This is a systematic review of peer-reviewed published literature, therefore ethics approval for the data used in this article was not required.

## Supplementary Material

czz044_Supplementary_Table
