# Supplementary material for: Investigating the process of evidence-informed health policymaking in Bangladesh: a systematic review
Source: Health Policy Plan. 2019 Jun 25;34(6):469–78. doi: 10.1093/heapol/czz044 (PMC6736329; doi:10.1093/heapol/czz044)
Supplement: czz044_Supplementary_Table [file czz044_supplementary_table.docx]

**Table 1:** Peer-Reviewed Literature Included in Review. Details about study type, policy issue, applied framework or theory and key findings.

| **Author, Year and Title** | **Study Type** | **Policy Issue** | **Theory or framework** | **Key findings** |
| --- | --- | --- | --- | --- |
| 1. (Mannan, 2003)   *“An Evaluation of the National Food and Nutrition Policy of Bangladesh.”* | SWOT analysis.  Qualitative study.  Strengths: Large sample size of 40 in-depth interviews and 6 focus group discussions, interview guides where designed around the ‘Strengths, Weaknesses, Opportunities and Threats’ framework. | Evaluation of the National Food and Nutrition policy approved in 1989 and revised in 1997. | SWOT analysis was performed to evaluate the national food and nutrition policy in Bangladesh. | 1. Policy was formulated through a multi-sectorial approach, involving experts in the country (agriculture, fisheries, ministry of health, ministry of women's and children's affairs). Both private and public sectors provided support during the policy formulation process.  2. Lack of government human and financial commitment to the policy was identified as a major weakness in implementing the policy.  3. Social mobilisation of the policy to increase awareness within the community was lacking. Some participants stated that nothing will happen in the field of nutrition if community participation is ignored.  4. Participants from the donor community identified weak and poor support from government officials and lack of strong coordination as other threats to the policy implementation. |
| 1. (Zafar et al., 2006)   *“Government–NGO collaboration: the case of tuberculosis control in Bangladesh.”* | Case-study.  Qualitative study.  Strengths: Results address the research question; multiple sources of data collected – in-depth interviews, focus groups discussions, peer-reviewed and grey literature and meeting minutes.  Weaknesses: Methods do not describe how data was collected or how it was analysed | Investigating how an appropriate public-private partnership can be established for a National TB Programme. | N/A | 1. NTP and NGOS had an MOU signed in 1995 that clearly defined each partners roles. Emphasis was given to the role NGOs had in mobilising civil society groups.  2. NGOs important in building an internal network of unified ideas to address a topic which then increases their ability to influence policy makers and global leaders.  3. Frameworks and guidelines designed by the govt enabled NGOs to have specific guidelines to follow during the delivery of training. |
| 1. *(Rounaq, 2007 )*   *‘‘Securing Maternal Health Through Comprehensive Reproductive Health Services: Lessons from Bangladesh Maternal and Infant Health in Diverse Setting.”* | Single Case-Study.  Qualitative study.  Strengths: Provides an historical narrative of how the reproductive health strategy has evolved in Bangladesh  Weakness: Does not describe methods for data collection clearly. | Reproductive health strategy over three decades (70s, 80s and 90s). | N/A | 1. Regulatory framework provided standards for private and public service provision.  2. Delays in implementation when govt and donor plans did not align.  3. Civil society groups influenced the sustained prioritisation of reproductive services.  4. Community participation was a key means to identify health needs and priorities, as well as mobilise political will.  5. First nationwide survey of maternal mortality in 2001 created objectives for a nationwide scale up of services. |
| 1. (Behague et al., 2009)   *“Evidence-based policy-making: the implications of globally-applicable research for context-specific problem-solving in developing countries.”* | Ethnographic qualitative study.  Sample size 52 (n=48) from 5 different countries – sufficient.  Thematic and comparative analysis used  Strengths: Clear research questions; sample size of 52 – key-informants selected from 5 different countries.  Weaknesses: Methods did not provide details about data analysis. | Maternal and newborn health. | ‘Actor Network Theory’ acknowledges how loosely connected scientific networks unite to translate their expert knowledge into materials that can influence wider audiences. (Latour, 2005) | 1. Research driven by national experts could potentially mitigate conflicts between external and national actors when implementing internationally driven EIPM.  2. Sub-reg  ional and regional researchers stated frustration about policies and programs that were designed at a national level and that weren’t locally relevant thus creating poor program implementation.  3. Policy windows for newborn health where created when international organisations started targeting newborn survival. Respondents provided mixed responses on this matter, some saw it as positive because donors and national governments were working as unified bodies while others thought it was negative because donors urged governments to concentrate on newborn health as a separate issue which conflicted with how local obstetricians viewed the issue.  4. Respondents explained that importing evidence-based policies derived from settings outside their own country undermines national expert’s experimental knowledge and the credibility of locally-generated solutions. |
| 1. (Rubayet et al., 2012)   *“Newborn survival in Bangladesh: a decade of change and future implications.”* | Single Case Study.  Mixed-methods study.  Strengths: Study used data from 2000 -2010 to demonstrate the trends in neonatal and maternal health. Document analysis of grey and peer reviewed literature was employed to map critical events.  Weaknesses: No weaknesses identified. | Neonatal and maternal health in Bangladesh. | N/A | 1. Signing of the Dhaka Resolution for Newborn Health marked commitment of the government to improving newborn health.  2. MoHFW were the leading organisation.  3. Wide range of high profile champions for newborn survival from many areas came together to advance newborn survival  4. Increased availability of funding for the program provided a window of opportunity and resources for implementation.  5. Release of the Lancet Neonatal Series in 2005. approx $US300 was granted to advance MNCH in Bangladesh. |
| 6.(Bennett et al., 2012)  *“Influencing policy change: the experience of health think tanks in low- and middle-income countries.*” | Case Study Analysis  Qualitative study.  Strengths: Conducted by researchers from the selected countries, who had a comprehensive understanding of the functions of the HPAI, but was not part of it.  Weakness: HPAIs studied were only those that opted to be involved, this could increase the bias within the results. | Effectiveness of Health Policy Analysis Institutes in supporting health policy development in LIMICs. | N/A | 1. Training emerged as a crucial mechanism that helped to strengthen the links between the institutes and policy makers.  2. Bangladesh received substantial core budgetary support from donors at start-up, but when these initial grants ended, the institutes found it difficult to find alternative funding sources to replace them.  3. Alteration in government decreased funding and support for institutes.  4. Funding most visible factor for decline of institute. |
| 7. (Larson et al., 2012)  *“Scaling up zinc treatment of childhood diarrhoea in Bangladesh: theoretical and practical considerations guiding the SUZY Project.”* | A single Case Study  Mixed-methods study..  Strengths: Comprehensive description of study purpose and clear description of the theoretical assumptions guiding the project design.  Weaknesses: No explanation of the different data collection or data analysis methods. | Scaling Up Zinc for Young Children (SUZY Project) | N/A | 1. High level of cohesion between policy communities consisting of MoHFW, paediatric academic leaders.  2. Advisory committee from within the MoHFW took the lead on increasing and implementing the scale up of zinc.  3. WHO/UNICEF reinforced the efficacy of Zinc treatment. Hosted a half day course reviewing the revised WHO/UNICEF guidelines.  4. Annual International Zinc Conference jointly sponsored by MoHFW and Icddr,b was organised and a biannual newsletter sent to 20 000 recipients. Project website with links to other sites addressing zinc or diarrhoea management used for evidence dissemination.  5. Clearly defined interventions were offered outlining the different aspects of the project that needed to be developed. |
| 8. (Burchett et al., 2012)  *“New vaccine adoption: qualitative study of national decision-making processes in seven low- and middle-income countries.”* | Comparative Case-study Analysis.  Qualitative study.  Strengths: Large sample size, 95 interviews in total – 13 from Bangladesh  Weakness: Study limitations discuss the changed priorities between GAVI and non GAVI eligible countries and how this could have impacted the study results | Analysis of ‘Expanded Programme on Immunization (EPI). | N/A | 1. In GAVI eligible countries EPI managers and staff played a central role in implementation.  2. In all countries the health minister played a central role. WHO considered as important stakeholder in GAVI countries.  3. Bangladesh intro of Hib vaccine assisted by the GAVI funded Hib Initiative – this group organised regional workshops and national consultative meetings.  International and national meetings noted as key events for setting international policy agenda.  4. Local disease burden data was important in three countries. It was noted that new vaccines would not be adopted if data did not indicate burden. |
| 9.(Pelletier et al., 2012)  *“Nutrition agenda setting, policy formulation and implementation: lessons from the Mainstreaming Nutrition Initiative.”* | Prospective Comparative Case-study.  Qualitative study.  Strengths: Methods clearly defined. Results presented clearly and answer the research question.  Weaknesses: No weaknesses identified. | Mainstreaming of Nutrition Initiative (MNI). | Shiffman and Smith’s 2007 Policy Prioritisation Framework. | 1. The sensational national media coverage resonated powerfully with senior bureaucratic officials and led to expert consultations on solutions  2. Appearance of clear policy alternatives was not crucial for initial agenda setting but crucial for maintaining political commitment and policy formulation.  3. Lack of cohesion between actors identified to result in significant delays in policy implementation.  4. Author acknowledges that political commitment and system wide commitment are two different components of policy formation – e.g. high level political speeches need to be matched with resource allocation. |
| 10.(Shiffman & Sultana, 2013)  *“Generating political priority for neonatal mortality reduction in Bangladesh.”* | Case study  Qualitative study.  Strengths: Sample size large Triangulation of data from different sources used to decrease the bias. Clear description of methods.  Weaknesses: No weaknesses identified. | Saving of Newborn Lives (SNL program) | Shiffman and Sultana’s policy prioritisation framework. | 1. Achieving MDG4 became a pillar for global and national institutions to achieve. Gov set up a national task force on the child and maternal survival MDGs in August 2007. National strategy for newborn survival was drafted by the MoHFW and a Bangladeshi research institute. Network of policy makers and researchers exerted influence over the programs run by other organisations.  2. In 1998 health and populations sector program promised greater health sector coherence after 2 decades of ineffectual government coordination and fragmentation of donor projects.  3. Focusing event occurred in 2003 called the Health Newborn Partnership that was convened in Dhaka. MoHFW opened the event and members from 31 different orgs attended.  4. Significant increase in funding of the programs –2000 Bangladesh one of the six priority countries Save the Children chose to fund newborn health strategies (budget of US$50 million.  5. 2007 BRAC began neonatal and child health project covering a population of 8 million people with a US$25 million budget from the Gates Foundation.  USAID began a US$15 million neonatal survival program in Bangladesh in 2009 |
| 11. (Ahmed et al., 2012)  *“Nutrition of children and women in Bangladesh: trends and directions for the future ”* | Cross Case-Study.  Mixed-methods study.  Strengths: Clearly outlines the aim of the study; describes data collection and data analysis methods.  Weaknesses: Due to the number of countries included in the study, results are generalised, making it difficult to provide advice about the most effective strategies. | Achievement of MDG 4 and MDG5a.  Reducing maternal and child mortality. | N/A | 1. Countries improved coordination, set priorities, developed long-term strategies and held firm to these commitments, demonstrating strong governance at the highest level, as well as a culture of accountability towards improved use of resources.  2. Improvements in governance enabled by a climate of relative political stability, which allowed policies to be maintained consistently over time and progressively improved.  3. LMICs used local data to support and drive policies |
| 12. (Uddin et al., 2013)  *“Introduction of New Vaccines: Decesion-making Process in Bangladesh.* International Centre for Diarrhoeal Disease Research Bangladesh.” | Single Case Study.  Qualitative Study.  Strengths: Interviews done over 3-month period. Documents reviewed from 2006.  Weaknesses: Data analysis not described in detail. | Mapping the process of the govt uptake of a new vaccines. | N/A | 1. Ultimate decision for vaccine introduction is with WHO and UNICEF while Ministry makes the guiding policy and guidelines.  2. Agreement required from researchers, ministry and international orgs for immunisations to be approved.  3. Key people involved in decision making include Director, Primary Health Care; Director General, DGHS; Joint secretary for PHC; Joint Secretary of the MoHFW and representative from ministry of finance.  4. Scientific evidence, particularly burden of disease data – research dissemination to high level policy officials was considered an important factor for the uptake of the Hib vaccine. |
| 13. (Balabanova et al., 2013)  *“Good Health at Low Cost 25 years on: lessons for the future of health systems strengthening."* | Comparative Case Study (Bangladesh, Ethiopia, Kyrgyzstan, Thailand and India).  Mixed-methods study.  Strengths: Multi-case study consisting of 5 countries; multiple sources of secondary and primary data used (statistical data relevant to health system, peer-reviewed literature and in-depth interviews); explanation of theories that underpinned data analysis was provided.  Weaknesses: Sample size for in-depth interviews omitted. | Analysis of 5 countries that have improved health and or health care systems without experiencing significant economic growth. | NPath dependency | 1. Stewardship implied, leadership from the government to formulate evidence based policies, design responsive services and to monitor results.  2. Funding local governments was an important part of success as a result of the large population.  3. Expansion of NGOs e.g. BRAC have made a major contribution to the training of health workers and funding of new initiatives – despite the improvements NGOs have made in health systems they have had less success in influencing policy change at a national level.  4. 1972 Bangladeshi constitution inserted legal right to health and created an environment open to voluntary and donor-led initiatives that complemented state programmes. - 1998 Bangladesh SWAp programme bought together 120 separate health programmes, reducing fragmentation and creating opportunities for scale up. |
| 14. (Baker et al., 2013)  *“Using an Evidence-Based Approach to Design Large-Scale Programs to Improve Infant and Young Child Feeding.”* | Comparative Case-Study.  Mixed-methods study.  Strengths: Study underpinned by socioecological model of behaviour change; uses different methodology to gather data from the four levels of the socioecological framework  Weaknesses: Provides approximate numbers for the in-depth interview sample sizes and does not clearly define the role of each included study group | UNICEF'S Global strategy for Infant and young child feeding | Socioecological model of change | 1. UNICEF leading organisation – linked the three countries together to share ideas.  2. WHO provided countries (Bangladesh, Vietnam and Ethiopia) guidelines to endorse IYCF policies.  3. Clear guidelines to define stunting, breast feeding, supplementary feeding definitions provided by the WHO.  4. Cost effective and available interventions provided for government, health workers and families to use. |
| 15.(El-Jardali et al., 2014)  “Capturing lessons learned from evidence-to-policy initiatives through structured reflection.” | Comparative Case-Study  Qualitative study.  Strengths: Clear study question. Appropriate data to address question IDI, FGD, observations and documents. Explained theory behind the framework Outcomes relate to question and represent the theory behind the methodology.  Weaknesses: No weaknesses identified. | Evaluate the effectiveness of local ‘Knowledge Translation Platforms’ (KTPs)  Multi-country study: Bangladesh, Nigeria, Burkina Faso, Cameroon, CAR, Ethiopia, Uganda, Sudan and Zambia | ‘Framework for assessing country-level efforts to link research to action’ – consists of 4 domains: The general climate for research use; the production of research that is relevant and synthesised appropriately; methods used to link research to action; and evaluation. (Lavis et al. 2006) | 1. Change of leadership within the government identified to slow the progression of KTPs.  2. KTPs found internal frame difficult to create due to limited ability to perform stakeholder mapping exercises, limited local data to create effective policy briefs and lacked skilled human resources.  3. Collaboration between KTPs and the government was seen as the most effective way for data to be used and programs to be scaled up.  4. Credible data seen as a major challenge for KTPs to influence policy change. Unwillingness for local research orgs and policymakers to share data made sourcing data difficult for KTPs.  5. Programs normally depended on funding from GAVI and partial funding from the MoHFW. |
| 16.(Khan et al., 2014)  “Smokeless tobacco control policies in South Asia: a gap analysis and recommendations.” | Comparative Case-Study.  Qualitative Study.  Strengths: Combination of document analysis and in-depth-interviews were used to conduct the case study; the methods clearly describe how the in-interview guides were designed and piloted, where the interview took place and how the participants were selected.  Weakness: Limited explanation surrounding the approach to data analysis; no details about the type of documents that were included in the study. | Smokeless Tobacco. | NWHOs MPOWER | 1. Lack of powerful political will identified as a barrier along with limited resources.  2. Limited public knowledge surrounding the harm associated with SLT and social acceptance for SLT identified as a limitation.  3. Influence of the large tobacco conglomerates and their active interference in effective policy formulation. |
| 1. (Bowser et al., 2014)   *“Global Fund investments in human resources for health: innovation and missed opportunities for health systems strengthening.”* | Comparative Case-Study.  Mixed-methods study.  Strengths: Mixed method analysis – clearly illustrates quantitative and qualitative data sources.  Weaknesses: Predominately related to the qualitative data about the expenditure of Global Fund investments. | Analysis of impact funding from the Global Fund to Fight AIDS, TB and Malaria had on human resources for health. | N/A | 1. Limited cohesion between government and donors – identified as being problematic for the long-term viability of the program.  2. Civil society was being mobilised to attend training sessions because of incentives – due to limited coordination the duplication of training initiatives was identified as an issue.  3. Not specifically about policy but is an important article for assessing factors that lead to the long-term sustainability of programs. |
| 1. (Ashraf et al., 2015)   *“Overview of a multi-stakeholder dialogue around Shared Services for Health: the Digital Health Opportunity in Bangladesh.”* | Observational descriptive study analysing outcomes of multi-stakeholder dialogues.  Qualitative study.  Weaknesses: The methods did not explain how data was collected and analysed to measure the success of the event.  Strengths: Clear description about the methods used to design and implement the MSD. | Scaling up the use of electronic and/or mobile communication technologies in healthcare. | N/A | 1. ehealth & mhealth considered important for sustaining women, children and adolescent health moving from MDG to SDG era.  2. Meeting headed by the MoHFW.  ‘MSD effective in articulating stakeholders’ priorities and determining critical areas of action’.  3. Neutral facilitation allowed the emergence of shared action plans and promotes ownership through active participation. |
| 1. (Jackson-Morris et al., 2015)   *“Multi-Stakeholder Taskforces in Bangladesh — A Distinctive Approach to Build Sustainable Tobacco Control Implementation.”* | Single Case-Study.  Qualitative Study  Strengths: Methods clearly articulated the process involved with identification of key-informants and the main themes of the interviews.  Weaknesses: No evidence that interview guides were piloted; key-informants were only from regional areas. Therefore, national perspectives were not included. | Examination of District Tobacco Control Taskforce. |  | 1. Taskforce integrated into meso, macro and micro levels of society.  2. MoHFW mandated to create a National Control Cell (NTCC) that aims to oversee and guide the implementation and enforcement of legislation.  3. The use of mobile courts to mobilise action at a civil level. Civil society groups were identified as important factors who ensured local representatives were accountable.  4. Interventions use existing program infrastructure that making them more cost-effective. |
| 1. (Walugembe et al., 2015) *“Utilization of research findings for health policy making and practice: evidence from three case studies in Bangladesh.”* | Exploratory case study  Qualitative study.  Strengths: Study based in Bangladesh only and used three different health topics to explore research utilization in Bangladesh. Using three different cases, provided authors with the ability to identify reoccurring themes. | Explorations of icddr,b’s research in policymaking processes. |  | 1. Stakeholder engagement before, during and after research is essential.  2. Input from researchers into women’s networks helped to shape their advocacy efforts resulting in the enactment of Domestic Relations Bill 2010.  3. Article suggested that using research to tie into other global norms can help to facilitate the uptake of research.  4. Appropriate planning for the dissemination of research was suggested as a recommendation to improve the bridging of the know-do gap. |
| 1. (Shroff et al., 2015)   *“Incorporating research evidence into decision-making processes: researcher and decision-maker perceptions from five low- and middle-income countries.”* | Cross-Case Study  Qualitative study.  Strengths: Strong methodology used; Case study from 5 countries that implemented a AHPSR project. Evaluation study included IDIs with key stakeholders. Evaluation studies were validated by other documents and peer reviewed literature.  . | H1N1 vaccination policy. I | ‘Framework for assessing country-level efforts to link research to action’ (Lavis et al).  ‘Two-Communities’ theory, researchers and policy-makers exist in two separate worlds with differing values and priorities. (Caplan, 1979). | 1. Policy briefs for H1N1 hugely valuable.  2. Pre-existing links between researchers and policymakers enhanced the perceived credibility of research and the project.  3. Project commended by policymakers for its dissemination strategy which involved a two-page summary brief which was decided on after consulting policy makers.  4. Increased pressure on govt to make prompt decision created policy window.  5. Findings reveal that policy briefs and policy dialogues alone are insufficient in enabling evidence informed decision making. |
| 1. (Hawkes et al., 2016)   *“Strengthening capacity to apply health research evidence in policy making: experience from four countries.”* | Comparative Case-Study.  Qualitative study.  Strengths: Data collected by research committee within the 5 research organisations that were being evaluated – findings were analysed by an independent researcher, multi-country study, data from Bangladesh included 20 in depth interviews and a document analysis; results and recommendations reported from the data are relevant and answer the research question.  Weaknesses: Methods lack information about the design of in-depth interviews and tolls that were used to evaluate researcher’s capacity to apply health research to policy making. | Evaluation of how civil society organisations (icddr,b) have developed the capacity of policy makers to use research evidence in their policy-cycles. | N/A | 1. The lack of a centralised site to share information with policy makers and understand their needs was a barrier to evidence uptake in many countries.  2. Research Policy Communication Cell was created to provide policy-makers within the MoHFW with access to training modules that can teach people how to access and use evidence.  3. Policy-makers identified that research outputs tend to be reports, research seminars or peer-reviewed publications few of which are seen as relevant to the policy making community. *'I feel shy to go to research dissemination programmes because I do not understand their findings especially the statistical part".* |
| 1. (Norton et al., 2016)   *“Exploratory study of the role of knowledge brokers in translating knowledge to action following global maternal and newborn health technical meetings.”* | Exploratory Study  Mixed-methods study.  Strengths: Study performed for 2 meetings with key stakeholders. Surveys used to collect data.  Weaknesses: Only 11 IDIs were conducted therefore difficult to capture the barriers and facilitators that each place experienced. | The role of multi-stakeholder meetings to improve knowledge translation efforts for the Maternal and Child Health Integrated Program.  Participants included academics, program managers, policymakers, health/medical service delivery and people involved with global advocacy. | Graham and Colleagues Knowledge-to Action-Framework. Focused on knowledge creation and the process of translating this new knowledge into action. (Field et al, 2014) | 1. Human interaction in the form of a MSD made it easier to create cohesion in a policy environment.  2. Uniting action to agree on terms can have positive impact on creating a policy window to push an agenda forward. |
| 1. (Teerawattananon et al., 2016)   *“The influence of cost-per-DALY information in health prioritisation and desirable features for a registry: a survey of health policy experts in Vietnam, India and Bangladesh.”* | Comparative Case Study  Qualitative study.  Strengths: Varity of stakeholders interviewed. 3 case studies selected to assess the question. The comparison of 3 different cases provided authors with the ability to identify reoccurring themes.  Weaknesses: Multi-case study featuring three countries. Sample small (n=32) considering three countries were included. | The barriers for the use of cost-effectiveness in LMIC and how this can be made more available. | World Health Organisation/Turning Research into Practice Framework (WHO/TRIP) | 1. Reported that attempts to increase support for health economic studies has little impact on the government.  3. Cost-effectiveness information is lacking in policy making.  All informants considered cost-effective studies as useful but stated often not available.  4. Interviewees stated that they are more interested in policy based on low costs rather than better outcomes. |
| *Abbreviations:* **AHPRS** – Alliance for Health Policy Research System; **CAR** – Central African Republic; **EPI** – Expanded Programme on Immunization; **Hib** – Haemophilus Influenzae; **IYCF** – Infant and Young Child Feeding; **KTP** – Knowledge Translation Platforms; **MNCH** – Maternal Newborn and Child Health; **MSD** – Millennium Development Goals; **PHC** – Primary Health Care; **SDG** – Sustainable Development Goals; **SLT** – Smokeless Tobacco Programme; **SWAp** – Sector-wide approach; **SWOT** – Strengths, Weaknesses, Opportunities, Threats; **UNICEF** – United Nations Children’s Fund; and **WHO** – World Health Organisation | | | | |
